# Supplementary material for: The Effect of Recycling on Wood-Fiber Thermoplastic Composites
Source: Polymers (Basel). 2020 Aug 5;12(8):1750. doi: 10.3390/polym12081750 (PMC7463641; doi:10.3390/polym12081750)
Supplement: Supplementary file 1 [file polymers-12-01750-s001.pdf]

Article

The effect of recycling on wood-fiber thermoplastic composites

Luísa Rosenstock Völtz, Irangeli Di Guiseppe, Shiyu Geng, and Kristiina Oksman*

Division of Materials Science, Department of Engineering Sciences and Mathematics, Luleå University of Technology, Luleå SE-971-87, Sweden; Luisa.voltz@ltu.se (L.R.V.); iradiyu@gmail.com (I.D.G.); shiyu.geng@ltu.se (S.G.)

* Correspondence: kristiina.oksman@ltu.se; Tel.:+46-920-493371

ANOVA AND TUKEY'S TEST ON FIBER ASPECT RATIO

Table S1. Fiber aspect ratio and Tukey's test after each recycling step.

No of passes	L/D
0	22±13 [A]
1	14±6 [B]
3	11±7 [B/C]
5	10±6 [B/C]
7	9±7 [C]
9	8±6 [C]

Marked with the same letter within the same column are not significantly different at 5% significant level based on ANOVA and Tukey's test.

SHEAR VISCOSITY

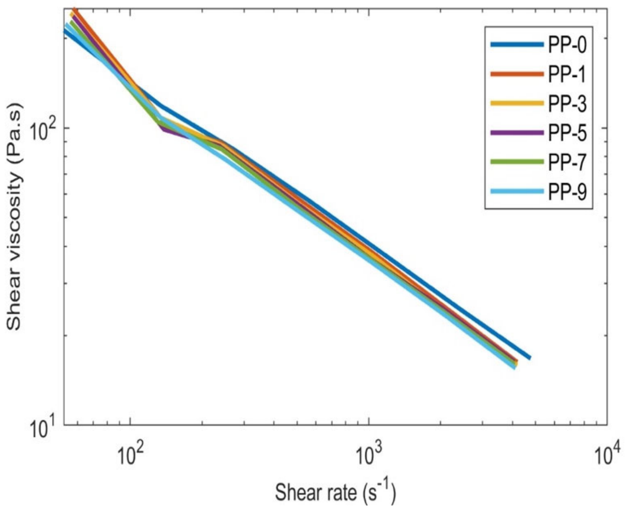

a)

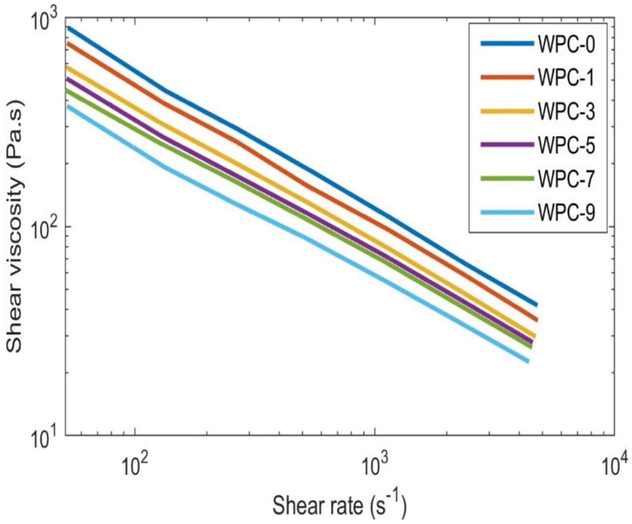

b)

Figure S1. Viscosity as a function of sheat rate after each recycling step. a) PP-0, PP-1, PP-3, PP-5, PP-7 and PP-9 and b) WPC-0, WPC-1, WPC-3, WPC-5, WPC-7 and WPC-9 at 230 °C

DIFFERENTIAL SCANNING CALORIMETRY

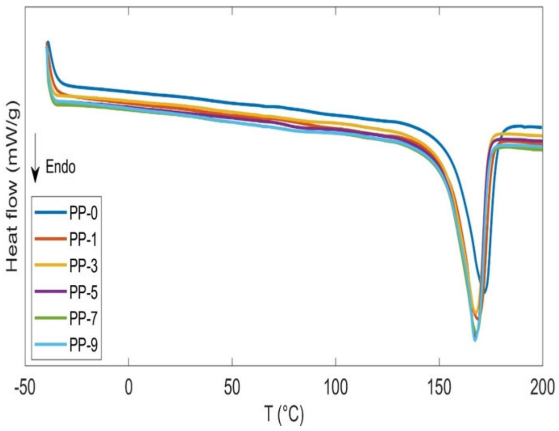

a)

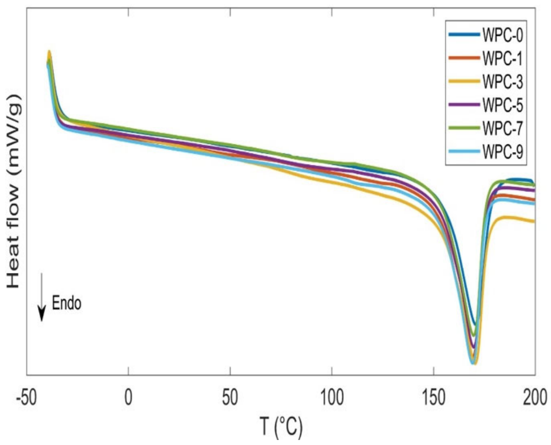

b)

Figure S2. Differential scanning calorimetry (DSC) graphs for the neat PP and WPC after each recycling step. a) PP-0, PP-1, PP-3, PP-5, PP-7 and PP-9 and b) WPC-0, WPC-1, WPC-3, WPC-5, WPC-7 and WPC-9.

THERMOGRAVIMETRIC ANALYSIS

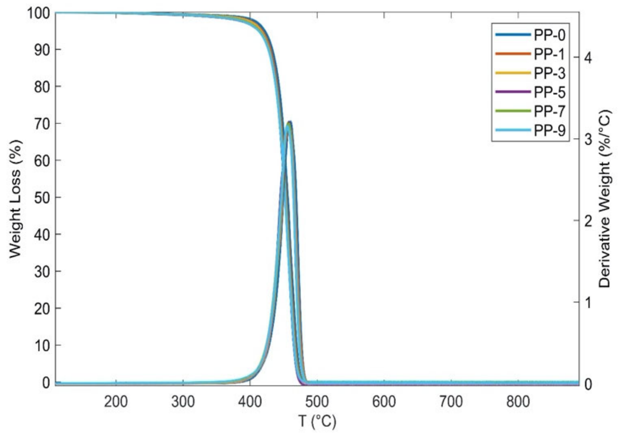

a)

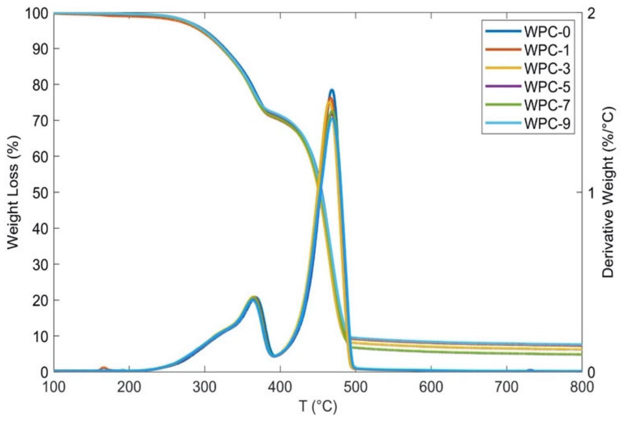

b)

Figure S3. Thermal degradation (TGA/DTG graphs) for PP and WPC after each recyscling step. a) PP-0, PP-1, PP-3, PP-5, PP-7 and PP-9 and b) WPC-0, WPC-1, WPC-3, WPC-5, WPC-7 and WPC-9.

SCANNING ELECTRON MICROSCOPE – FRACTURE SURFACES

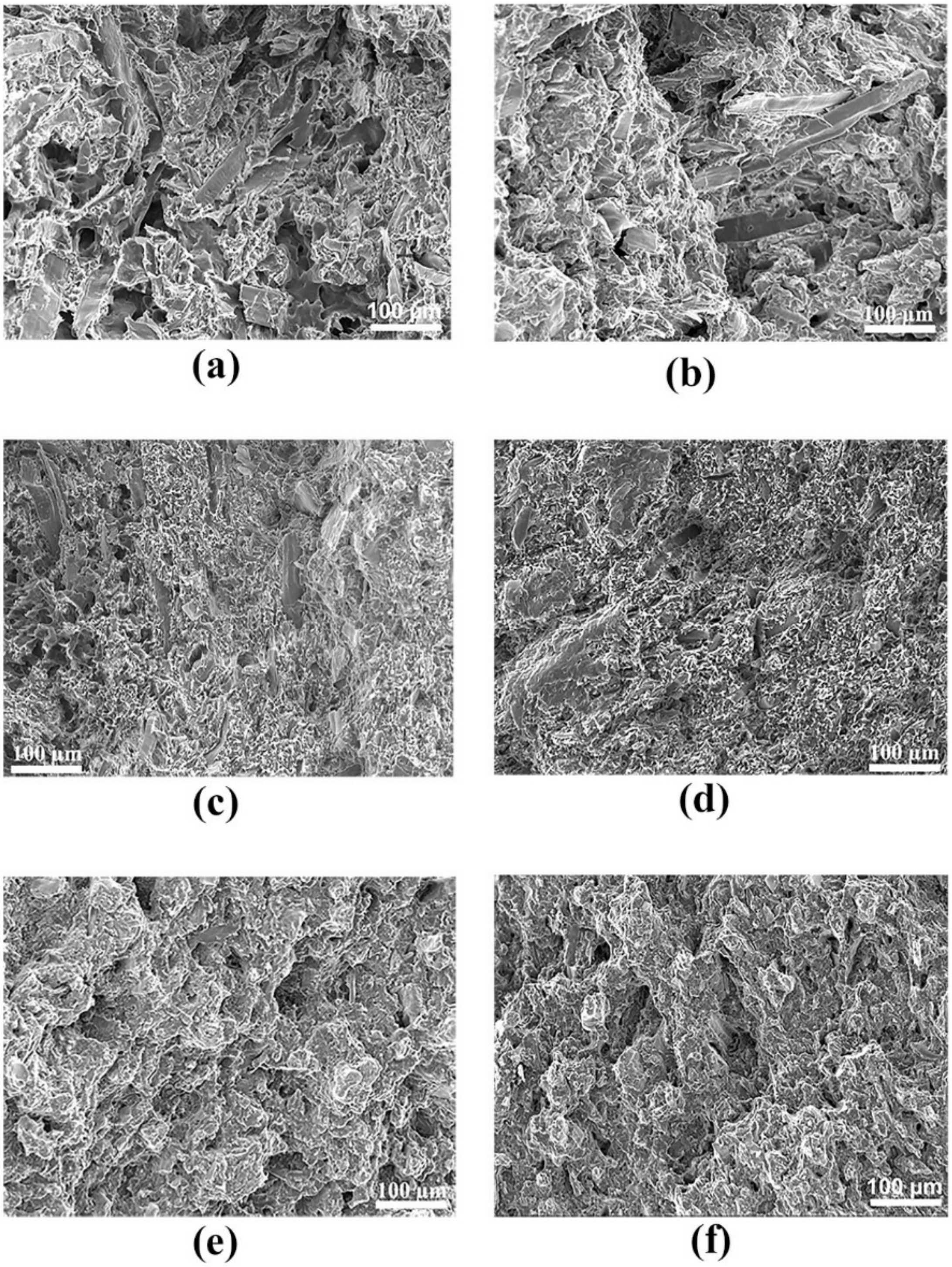

Figure S4. Fracture surfaces of WPC after each recycling step showing that interaction between the MAPP and WPC is improved during repeated processing. (a) WPC-0, (b) WPC-1, (c) WPC-3, (d) WPC-5, (e) WPC-7 and (f) WPC-9.
